# Listening to Preferred Music Improved Running Performance without Changing the Pacing Pattern during a 6 Minute Run Test with Young Male Adults

**DOI:** 10.3390/sports8050061

**Published:** 2020-05-11

**Authors:** Nidhal Jebabli, Urs Granacher, Mohamed Amin Selmi, Badriya Al-Haddabi, David G. Behm, Anis Chaouachi, Radhouane Haj Sassi

**Affiliations:** 1 Health and Movement (2SHM) Laboratory, Sport Sciences, High Institute of Sport and Physical Education of Kef, University of Jendouba, Le Kef 7001, Tunisia; jnidhal@gmail.com; 2 Higher Institute of Sport and Physical Education, Ksar-Said, University of Manouba, Tunis 2010, Tunisia; 3 Division of Training and Movement Sciences, University of Potsdam, 14469 Potsdam, Germany; 4 Tunisian Research Laboratory “Sport Performance Optimization”, National Center of Medicine and Science in Sports (CNMSS), Tunis 1003, Tunisia; selmi.med.amin@gmail.com (M.A.S.); chaouachi_anis@hotmail.com (A.C.); 5 Physical Education Department, College of Education, Sultan Qaboos University, Muscat 123, Oman; bhaddabi5@gmail.com (B.A.-H.); radhouane.hsassi@gmail.com (R.H.S.); 6 School of Human Kinetics and Recreation, Memorial University of Newfoundland, St. John’s, NL A1C 5S7, Canada; dbehm@mun.ca; 7 Sports Performance Research Institute New Zealand, AUT University, Auckland 0632, New Zealand

**Keywords:** RPE, work-rate distribution, blood lactate, aerobic exercise

## Abstract

Several studies have investigated the effects of music on both submaximal and maximal exercise performance at a constant work-rate. However, there is a lack of research that has examined the effects of music on the pacing strategy during self-paced exercise. The aim of this study was to examine the effects of preferred music on performance and pacing during a 6 min run test (6-MSPRT) in young male adults. Twenty healthy male participants volunteered for this study. They performed two randomly assigned trials (with or without music) of a 6-MSPRT three days apart. Mean running speed, the adopted pacing strategy, total distance covered (TDC), peak and mean heart rate (HRpeak, HRmean), blood lactate (3 min after the test), and rate of perceived exertion (RPE) were measured. Listening to preferred music during the 6-MSPRT resulted in significant TDC improvement (Δ10%; *p =* 0.016; effect size (ES) = 0.80). A significantly faster mean running speed was observed when listening to music compared with no music. The improvement of TDC in the present study is explained by a significant overall increase in speed (main effect for conditions) during the music trial. Music failed to modify pacing patterns as suggested by the similar reversed “J-shaped” profile during the two conditions. Blood-lactate concentrations were significantly reduced by 9% (*p* = 0.006, ES = 1.09) after the 6-MSPRT with music compared to those in the control condition. No statistically significant differences were found between the test conditions for HRpeak, HRmean, and RPE. Therefore, listening to preferred music can have positive effects on exercise performance during the 6-MSPRT, such as greater TDC, faster running speeds, and reduced blood lactate levels but has no effect on the pacing strategy.

## 1. Introduction

Pacing refers to the pattern in which athletes distribute work and energy throughout an exercise task [1]. It is well-documented that pacing strategies [1,2,3] and the choice of the appropriate pacing strategy have an impact on performance. Success in short-duration performances [4] and in middle- and long-distance running events can be influenced by the pacing strategy [5].

Coaches and researchers have described a variety of pacing strategies such as the negative, all-out, positive, even, parabolic-shaped (U, J, reverse J), and variable pacing strategies [1]. Previous studies showed that exercise performance and pacing strategies depend on specific factors such as knowledge of the endpoint [6], performance level, competitors [7], and music [8,9].

Many studies have shown the beneficial effects of music on sport-specific performance, particularly during aerobic events [9,10,11]. The use of music as an ergogenic aid may enhance performance by influencing exercise intensity (i.e., running speed and heart rate) and rating of perceived effort (RPE) [9,11,12]. In addition, Karageorghis et al. [12] reported that the careful application of music can lead to a number of benefits that include lower perceived exertion (RPE), greater energy efficiency, and faster time trial performances. Maddigan et al. [9] reported music-induced increases in running duration, breathing frequency, and respiratory exchange ratio, a decrease in RPE, as well as a faster heart rate recovery compared to the control condition. They rationalized that music provided a divergent stimulus that modified central nervous system control of volitional fatigue. Furthermore, Bigliassi et al. [13] postulated that selected music characteristics such as rhythm, familiarity, and music selection have the potential to influence exercise performance. Cole and Maeda [14] found that listening to preferred instead of non-preferred music had a greater positive effect on the 12 min Cooper test in young healthy females but not in males.

RPE as a marker of subjective perception of effort during exercise is thought to be part of a regulatory motor program that incorporates a number of physiological parameters and psychological and affective components [15]. Accordingly, Edworthy and Warring [11] reported that listening to fast music during exercise may lower RPE by directing attention away from physical fatigue toward music, which seems to allow athletes to sustain higher exercise intensity. Lima-Silva et al. [16] suggested that the manipulation of external cues, such as music, is able to modify RPE during exercise and consequently it may influence the adopted pacing strategy and performance level. Numerous studies have examined the effects of music on RPE and cardiorespiratory variables such as heart rate, arterial pressure, and oxygen uptake [3,8,17]. However, there is a paucity of studies that have examined the effects of music and its relationship with particularly anaerobic metabolism such as blood lactate concentration [9,18]. Maddigan et al. [9] reported a near-significant increase (*p* = 0.08) in the blood lactate concentration (12.5%) with the music and running conditions. They suggested that music contributes to greater intensity of effort in relationship with the increase of psycho-physiological parameters. In this context, Borg [17] reported that heart rate and blood lactate together could predict RPE more precisely than either variable taken alone.

The choice of the type and intensity of exercise are other factors that may influence the effects of music on fitness performance. Van Dyck and Leman [19] suggested that the ergogenic effect of music declines with increasing intensity levels. They stated that “at high-intensity levels, physiological cues seem to dominate the exerciser’s processing capacity, while at more moderate levels; both musical and physiological cues can be processed in parallel.” Indeed, Elliott et al. [20] reported that music could have a positive effect especially during low-to-moderate intensity exercise (i.e., below the anaerobic threshold). Although numerous studies have examined the effects of music by using low-to-moderate exercise at constant work load and high-intensity exercise [11,21], little is known on the distribution of self-selected work rate during self-paced exercise [8,16]. Listening to music can increase the distance covered during a 15 min self-paced maximal run on a treadmill [10] and improve cycling speed during the first 3 km of a 10 km cycling time trial [8]. Most studies that examined the effects of music on pacing have used externally imposed music [8,16], but a few studies have examined the effects of listening to preferred music. Moreover, the majority of studies have used cycling and running on a treadmill to examine the effect of music on performance [8,16,21,22]. However, to our knowledge, there are no studies that evaluated the effects of preferred music on both pacing strategy and physical performance during outdoor self-paced running tests.

Therefore, the aim of this study was to examine the effects of listening to preferred music on various performance (i.e., total distance covered (TDC), running speed, pacing) and physiological measures (i.e., blood lactate concentration, heart rate) in young healthy male adults. With reference to Barwood [10] and Atkinson et al. [8], we hypothesized that listening to preferred music improves TDC, running speed, and pacing with concomitant changes in physiological parameters (e.g., blood lactate).

## 2. Materials and Methods

### 2.1. Subjects

Based on a medium-sized effect of listening to music on exercise performance reported by Maddigan et al. [9], an a priori power analysis was calculated with G*Power (Version 3.1.9.2, University of Kiel, Kiel, Germany) using the f test family (repeated measures, within factors) and 2 experimental conditions (music versus control). The analysis revealed that a total sample size of *N* = 19 would be sufficient to find significant and medium-sized effects of condition (effect size *f* = 0.6, *α* = 0.05) with an actual power of 0.80. Thus, 20 healthy male physical education students aged 22 ± 1.3 years (body mass: 75.1 ± 7.7 kg; body height: 1.8 ± 0.3 m; body mass index [BMI]: 23.2 ± 1.4 kg·m^−1^, estimated VO_2_ max: 50.4 ± 3.4 mL·min^−1^·kg^−1^) volunteered to participate in this study. Study participants were active in various sports such as combat sports, basketball, football, and athletics. On average, participants were physically active 16 h·wk^−1^. Participants regularly participated in physical education classes including ball games, swimming, athletics, gymnastics, combat sports, music, and dance. None of the subjects reported any current or ongoing neuromuscular diseases or musculoskeletal injuries specific to the ankle, knee, or hip joints. They were asked not to take any dietary or performance supplements that may have affected their performance during testing. Participants performed tests at the same time of day with two experimental conditions in a counterbalanced order separated by at least 72 h. Participants were asked to maintain similar diets 24 h before each trial, which was established from the food diary. Participants were then asked to avoid caffeinated products and alcohol 24 h prior to each session and to avoid physical activity 48 h before each session.

Written informed consent was obtained from all participants after verbal and written explanation of the experimental design and potential risks of the study. The present study was conducted according to the latest version of the Declaration of Helsinki and the protocol was fully approved by the local Ethics Committee of our university (UR13JS01) before the commencement of the tests. All participants were fully familiarized with the applied procedures. They could withdraw from the study at any time of the experiment without providing reason.

### 2.2. Procedures

Participants were required to participate in three test sessions. All tests were completed within a two-week period and each test was separated by at least 72 h. Before the tests started, subjects completed a standard 15 min period of warm-up, including 3–5 min of light jogging, lateral displacements, dynamic stretching, and jumping. During the first session, all subjects performed the Vameval test to assess their maximal aerobic speed (MAS) and estimate VO_2_ max (maximal aerobic capacity). During the two last sessions, subjects performed in a random order, a 6 min self-paced run test under two different conditions: listening to preferred music during the test or without music (WM). For each test trial, participants had to run for 6 min as fast as they could to cover the longest possible distance. The participants were asked to select their preferred music, for when they participate in aerobic exercise. For the music condition, all preferred tracks were played using the same device with the same mp3 player, keeping the same moderate volume for all participants (around 50% of maximum).

### 2.3. Preferred Music

The music played for each participant was self-selected. Indeed, the participants were asked to select a minimum of 10 min of music, specifically those with which they felt more inclined to exercise aerobically. All songs chosen by the participants were characterized by a fast rhythm (120–140 beats·min^−1^). One investigator quantified the average tempo for each song selected to verify that it was within the required tempo range using “Free BPM Detector” software. Music was played from an mp3 player with headset during the music trial with comfortable and moderate volume intensity. During the no music trial, the mp3 player and headset were used as well, but no music was played.

### 2.4. Measurements

#### 2.4.1. Vameval Test

The Vameval test begins with a running speed of 8.5 km·h^−1^ and increases by 0.5 km·h^−1^ every minute until exhaustion. The participants adjusted their running speed to the auditory signals at 20 m intervals, delineated by marks placed at 20 m intervals along a 400 m athletics track. The test ended when the subject could no longer maintain the required running speed dictated by the audio beep, for two consecutive occasions. The measured heart rate at the end of the test must be similar to the predicted maximal heart rate, which was estimated according to the formula of Tanaka et al. [23]. MAS corresponds to the last-stage speed completed [24].

#### 2.4.2. 6 Min Self-Paced Run Test

The purpose of the 6 min self-paced running test (6-MSPRT) was to cover the longest distance within 6 min [25]. Before the test started, participants performed a standard 10 min warm-up that included walking, running, and dynamic stretching exercises. Thereafter, participants rested for three minutes in an upright position before the 6 min run test started. During the two test trials, participants were asked to cover the longest possible distance on a 400 m outdoor athletics track. As a reference, 20 cones were placed every 20 m on the track to measure the distance covered by each participant every 30 s. Running speed was then calculated for these same intervals. The distribution of work rate during the test was assessed by measuring running speed every 30 s. Total distance covered was also determined (m). Heart rate was continuously recorded during the two last trials of the test using a heart rate monitor (Polar team 2, New York, NY, USA). Both peak heart rate (HRpeak) and mean heart rate (HRmean) values were observed during the 6-MSPRT. Three minutes after each test trial, blood lactate concentration was measured by using a Lactate Monitor (lactate pro 2, Akray, Japan). RPE was registered with the help of the 6–20 Borg scale (Borg, 1985).

### 2.5. Statistical Analyses

Data were expressed as means and standard deviations (SD). Normality of data was assessed and confirmed using the Kolmogorov–Smirnov test. The paired Student’s t-test was used to compare total distance covered over the 6 min run test. In addition, RPE, HRpeak, HRmean, and blood lactate concentrations were recorded at post-tests. A two-way ANOVA (two conditions (music vs. control) × 12 times (every 30 s over the 6 min run)) was computed for 30 s running speed intervals. Effect sizes (ESs) were computed to indicate the magnitude of the findings (Cohen’s d). ESs were classified according to Hopkins et al. [26] as trivial ≤0.2, small >0.2–0.6, moderate >0.6–1.2, large >1.2–2.0, and very large >2.0 magnitudes. The level of significance was set at *p* ≤ 0.05. All analyses were carried out using SPSS 16 for Windows (SPSS, version 16 for Windows. Inc., Chicago, IL, USA).

## 3. Results

**Figure 1:**
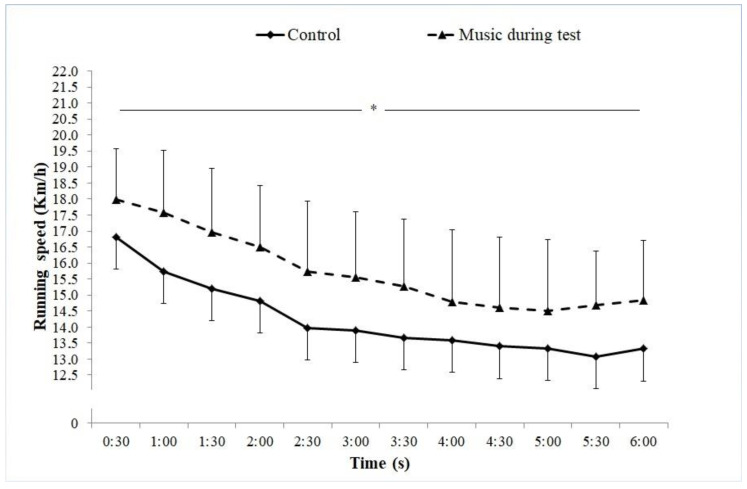
The overall effect of listening to preferred music on speed during the 6 min run test. * Significant main effect of condition (*p* < 0.05).

There was a significant increase in TDC with music compared with control (1566.55 ± 179.73 m vs. 1422.35 ± 178.76 m; Δ10%; *p =* 0.016; ES = 0.80) during the 6-MSPRT. Figure 1 represents the effects of listening to preferred music vs. control on speed at each 30 s during a 6 min run test. No significant condition × time interactions were observed (*p* = 0.539; ES = 0.010). However, our results showed a significant (*p* = 0.012; ES = 0.154) main effect of condition (between the two sessions). Pacing strategies were not modified by listening to music since participants adopted the same reversed “J-shaped” profile without significant increases in speed at the end of the 6-MSPRT (no end spurt) in the two conditions (Figure 1).

No significant pre- to post-test 6-MSPRT differences were recorded for HRpeak, HRmean, and RPE during the two trials (Table 1). However, a significant decline in blood lactate concentration was observed with music compared to the control condition (Δ9%, *p =* 0.006, ES = 1.09). There were no significant HR differences between the two conditions during the (6-MSPRT).

## 4. Discussion

The purpose of this study was to examine the influence of listening to preferred music during a 6 min self-paced run test on speed distribution (pacing), blood lactate, HRpeak, HRmean, and RPE. The main findings of this investigation were that preferred music, compared to no music, improved TDC with an increase in running speed throughout the 6-MSPRT. A significant decrease in blood lactate at 3 min after the end of the test was also observed. A reversed “J-shaped” speed profile during the test was observed in the two conditions. Moreover, no significant effects of listening to music on the HRpeak, HRmean, and RPE were found.

Most previous studies that have examined the effects of music on performance have used submaximal constant-intensity exercise, which does not faithfully reproduce what athletes do in most sporting events, particularly, the pacing strategies they can adopt. In contrast to research that employed laboratory exercise based on cycling or running-on-treadmill protocols, the current study used an outdoor self-selected running-rate exercise to better reflect real training and competition situations making the present findings useful for further applications.

In the present study, TDC increased significantly during the music condition (∆10%; *p =* 0.015) compared to the no-music trial. This result is in line with a number of studies that have shown the beneficial effects of music on aerobic performance (>3 min) by enhancing submaximal exercise performance duration [9,21], total distance covered [10,11], or cycling time trial [8]. The improvement of TDC in the present study is explained by a significant overall increase in speed (main effect for conditions) during the music trial. This result is in agreement with that reported by Edworthy and Waring [11], who showed that during a 10 min self-paced treadmill exercise, participants realized higher treadmill speed when listening to music than without. Similarly, Atkinson et al. [8] showed that the improvement of a 10 km cycling time was essentially due to an increase in speed during the first 3 km of the time trial.

In the current study, the characteristic of a reversed “J-shaped” profile of speed with no end spurt was observed during the 6-MSPRT in both test conditions. In other words, participants ran faster at the start of the exercise, after which speed declined until the end of the exercise without any significant increase of speed at the last minutes of the 6-MSPRT (no end spurt). This result is not in line with that reported by Atkinson et al. [8], who observed the characteristics of a U-shaped profile of speed during the time trial in the music condition but not in the no-music trial. The differences between the two types of strategies adopted in the two studies could be due to the different exercise protocols used (10 km cycling time trial versus 6-MSPRT). Noakes et al. [1] hypothesized that listening to music could affect the pacing strategy and thereby physical performance by keeping the cadency along the task by possibly minimizing performance decrements in some moments of the activity, which is the case in the current study. In fact, it seems that the participants in this study could have used the music to dissociate feelings of fatigue toward the end of the 6-MSPRT as shown by the increase of TDC and speed in the music condition. In addition, Lima-Silva et al. [16] suggested that listening to music may draw the intentional focus away from internal sensations of fatigue to thoughts about the external environment. As suggested by Tucker et al. [5], the significant increase of speed during the music condition, such as the case in this study, could be an indication of the maintenance of a reserve capacity allowing the speed to increase secondary to an increase in central neural drive to exercising muscles. Indeed, Bigliassi et al. [13] reported that a synchronization mechanism, which refers to the predisposition of a subject to synchronize movement with musical rhythm, shows the ability of the brain to subconsciously synchronize specific cyclic movement during running or cycling with the beat per minute of music. Furthermore, Schneider et al. [27] suggested that music tempo influences physiological processes through the brain’s regulation of locomotion, cardiovascular control, and sensory input.

In the present study, this suggestion was supported by blood lactate production measured 3 min after the end of the exercise, which was significantly lower during the exercise with music than in the control condition. This result is in accord with Szmedra and Bacharach [18], who showed that following a treadmill exercise at 70% VO_2_ max with music, blood lactate was significantly lower (22.5%) when compared to the no-music condition. These researchers suggested that the decrease of blood lactate in the music condition could be due to the relaxing effect of music, which induces a decrease of muscle tension, and thereby increases blood flow and lactate clearance, while reducing lactate production in active muscles. It seems that synchronization of movement with musical rhythm enables participants to perform more efficiently, resulting in increased levels of work output. In fact, Fritz et al. [28] showed that participants were able to apply a comparable amount of work using less oxygen during musical agency than during passive listening. Likewise, Bacon et al. [29] reported that participants who cycled in time to music required less oxygen to perform the same amount of work as compared to cycling with asynchronous music. They suggested that music may provide temporal cues that make athletes’ energy use more efficient. Similarly, Fritz et al. [28] suggested that music could allow participants to create “virtual goals” with anticipatory endpoints that allow them to adjust and monitor the extent and the timing of their movements more effectively. However, current data were not in agreement with previous studies that have shown that at the end of exercise, blood lactate concentrations were not significantly different between music and no-music conditions [30,31]. These differences could be due to the choice of the music tempo, the population studied, and the nature of the exercise protocols [13]. In agreement with our observation, Karageorghis et al. [12] revealed that tempo is the most important factor for determining motivation provided through listening to music and its effect on physical performance. Therefore, further research on the effect of music during exercise on metabolic cost, neuromuscular and metabolic efficiency, and stress hormones should be studied.

Although, a significant decrease in blood lactate concentrations measured 3 min after the end of the exercise was observed in the present study during music condition, there were no significant differences in HRmean and HRpeak during the two test trials. The improvement of TDC through an increase of running speed in the music condition seems not to be the consequence of a direct effect of music on heart rate (HR) responses. There have been conflicting results reported for the effect of music on HR responses. Lee and Kimmerly [30] showed that listening to fast music resulted in a faster self-selected running speed and higher exercise HR during a 20 min of self-paced maximal treadmill exercise. Likewise, Atkinson et al. [8] reported that the improvement of 10 km cycling time was accompanied by a significant increase of HRmean. However, Dyer and McKune [31] indicated that listening to music did not affect performance and HR during a 20 km cycling in well-trained cyclists. Yamashita et al. [22] found that the effect of music was not significant when exerting at intensities of 40% and 60% of maximal aerobic power and concluded that differences in autonomic nervous system activity were affected only by the exercise intensity and not by music. Results of this study showed that participants increased their running speeds without increasing their HRmean and HRpeak, which could indicate that the cardiovascular system works more efficiently with music than without. Although HR is considered as the most practical parameter of the heart’s work during exercise, it is not the only indicator of the functions of cardiovascular system. Further research is needed to better clarify the effect of music on cardiac output and stroke volume. On the other hand, results of the current study showed no significant difference in RPE between test conditions despite the significant increase in running speed and TDC during the music trials. This is in accordance with several studies that have shown that listening to music increases performance without any significant change in RPE [11,16,30]. However, Atkinson et al. [8] reported that both mean RPE and cycling speed were significantly higher throughout the entire distance of a 10 km time trial with music than without. Dyer and McKune [31] showed that performance and RPE were not significantly different during a 20 km cycling time trial in well-trained cyclists. A limitation of the current study is that we recorded the RPE only at the end of exercise, which makes it difficult to compare with other studies that recorded RPE throughout the exercise. The fact that listening to music increased running speed and TDC without influencing RPE could support that music distracted participants from the higher intensity of exercise. In other words, RPE data recorded in the present study may be considered as attenuated taking into account the higher speed performed in the music condition compared to no-music trial. Eston [32] stated that “RPE involves the collective integration of afferent feedback from cardiorespiratory, metabolic, and thermal stimuli and feedforward mechanisms to enable an individual to evaluate how hard or easy an exercise task feels at any point in time.” Results of the current study showed that RPE and HR did not change significantly despite the significant improvement of TDC and the significant decrease of the exercise blood lactate concentrations. Borg et al. [17] indicated that the combination of HR and blood lactate predict RPE more accurately than either variable taken alone. Based on the fact that performance in endurance running, such as the 6 min run exercise, is mainly related to a high cardiac output (HR × stroke volume), a high oxygen delivery to working muscles, the ability to sustain a high percentage of VO_2_ max for long periods of time, and the ability to move efficiently, as proposed by Foster et al. [3], it seems that RPE is more influenced by afferent feedback from the cardiovascular system (HR) rather than from metabolics (blood lactate concentration).

## 5. Conclusions

In light of these study findings, listening to preferred music improved 6 min self-paced maximal exercise performance through an increase in TDC and decreased blood lactate concentration without any change in HR and RPE. In addition, the pacing strategy during this type of exercise was unaltered by listening to music as suggested by the same reversed “J-shaped” profile observed in the two test conditions. However, participants appeared to sustain higher running speed while listening to music without consciously feeling this higher intensity than during the no-music condition. The absence of significant differences in HR between the two conditions despite the increase in running speed could mean an improvement in cardiovascular efficiency. Further understanding is needed to establish how RPE is regulated during self-paced exercise and its relationship to pacing strategy while listening to preferred music.

As a practical consequence of our findings, we recommend coaches and strength and conditioning specialists to allow their athletes to listen to preferred music during all-out endurance tests. Thus, the likelihood increases that the tested athletes realize their full performance potential during all-out tests.

## Figures and Tables

**Table 1:** The effects of listening to preferred music vs. control on heart rate, and rating of perceived exertion during and after the 6 min run test.

	Control	Music	*p* Value	ES
HRpeak (beat/min)	191.3 ± 8.2	190.4 ± 8.5	0.619	-
HRmean (beat/min)	178.7 ± 9.5	179.1 ± 7.1	0.791	-
RPE post-test	16.9 ± 1.3	17.3 ± 1.5	0.379	-
Lactate (mmol/L)	17.3 ± 1.4	15.9 ± 1.3	0.006 *	1.09

Data were expressed as means ± standard deviations; ES: effect size, RPE post-test: Rating of perceived exertion after the test, HR: heart rate; * Significant difference between music and control conditions (*p* < 0.01).
